# ETF-QO Mutants Uncoupled Fatty Acid β-Oxidation and Mitochondrial Bioenergetics Leading to Lipid Pathology

**DOI:** 10.3390/cells8020106

**Published:** 2019-01-31

**Authors:** Suphannee Chokchaiwong, Yung-Ting Kuo, Sung-Po Hsu, Yi-Ching Hsu, Shih-Hsiang Lin, Wen-Bin Zhong, Yung-Feng Lin, Shu-Huei Kao

**Affiliations:** 1 Ph.D. Program in Medical Biotechnology, College of Medical Science and Technology, Taipei Medical University, Taipei 11031, Taiwan; d609105003@tmu.edu.tw (S.C.); yflin@tmu.edu.tw (Y.-F.L.); 2 School of Medical Laboratory Science and Biotechnology, College of Medical Science and Technology, Taipei Medical University, Taipei 11031, Taiwan; yichinghsu@tmu.edu.tw (Y.-C.H.); gege1202@gmail.com (S.-H.L.); 3 Department of Pediatrics, School of Medicine, College of Medicine, Taipei Medical University, Taipei 11031, Taiwan; pedkuoyt@s.tmu.edu.tw; 4 Department of Pediatrics, Shuang Ho Hospital, Taipei Medical University, New Taipei 23561, Taiwan; 5 Department of Physiology, School of Medicine, College of Medicine, Taipei Medical University, Taipei 11031, Taiwan; sphsu@tmu.edu.tw (S.-P.H.); wbzhong@gmail.com (W.-B.Z.); 6 Graduate Institute of Medical Sciences, College of Medicine, Taipei Medical University, Taipei 11031, Taiwan

**Keywords:** multiple acyl-CoA dehydrogenase deficiency, electron-transfer flavoprotein dehydrogenase, electron-transfer flavoprotein-ubiquinone oxidoreductase, mitochondrial dysfunction, lipid droplet accumulation

## Abstract

The electron-transfer flavoprotein dehydrogenase gene (*ETFDH*) that encodes the ETF-ubiquinone oxidoreductase (ETF-QO) has been reported to be the major cause of multiple acyl-CoA dehydrogenase deficiency (MADD). ETF-QO is an electron carrier that mainly functions in mitochondrial fatty acid β-oxidation and the delivery of electrons to the ubiquinone pool in the mitochondrial respiratory chain. A high frequency of c.250G>A has been found in Taiwanese patients with late-onset MADD. We postulated that the *ETFDH* c.250G>A mutation may concomitantly impair fatty acid β-oxidation and mitochondrial function. Using MADD patient-derived lymphoblastoid cells and specifically overexpressed *ETFDH* c.92C>T, c.250G>A, or coexisted c.92C>T and c.250G>A (c.92C>T + c.250G>A) mutated lymphoblastoid cells, we addressed the genotype-phenotype relationship of *ETFDH* variation in the pathogenesis of MADD. The decreased adenosine triphosphate synthesis, dissipated mitochondrial membrane potentials, reduced mitochondrial bioenergetics, and increased neutral lipid droplets and lipid peroxides were found in the MADD patient-derived lymphoblastoid cells. Riboflavin and/or coenzyme Q10 supplementation rescued cells from lipid droplet accumulation. All three mutant types, c.92C>T, c.250G>A, or c.92C>T + c.250G>A, had increased lipid droplet accumulation after treatment with palmitic acid. These results help to clarify the molecular pathogenesis of MADD as a result of the high frequency of the *ETFDH* c.250G>A and c.92C>T mutations.

## 1. Introduction

Multiple acyl-coenzyme A (CoA) dehydrogenase deficiency (MADD, MIM#231680), also known as glutaric aciduria type II, is an inherited, autosomal recessive disorder [1]. MADD presents with a broad spectrum of symptoms, including hypotonia, hypoglycemia, recurrent rhabdomyolysis, cardiomyopathy, polycystic kidneys, and symmetric warty dysplasia of the cerebral cortex, encephalopathy, leukodystrophy, and lipid storage myopathy [2]. The clinical manifestations in MADD are highly variable and related to the onset period. In the late-onset type, MADD may manifest beyond the neonatal stage as isolated muscle weakness and myofibril destruction because of intracellular lipid deposits [3].

MADD primarily results from the absence and/or inactivity of either electron-transfer flavoprotein (ETF) or electron-transfer flavoprotein ubiquinone oxidoreductase (ETF-QO, also called ETFDH) [1,4]. ETF and ETF-QO are functionally associated; they both conduct electron transfer from flavin adenine dinucleotide (FAD)-containing acyl-CoA dehydrogenases for fatty acid β-oxidation to the ubiquinone pool for mitochondrial respiration [5]. ETF-QO couples electron transfer between acyl-CoA (as the electron donor) and ubiquinone (as the electron acceptor). Many patients with MADD have mutations in one of three genes (*ETFA*, *ETFB*, and *ETFDH*) that encode ETF-α, ETF-β, or ETF-QO. In the last 10 years, the genotypes for 440 late-onset MADD patients were reported, and it was found that more than 90% of late-onset MADD patients harbored recognizable *ETFDH* gene mutations [1,6,7,8]. More than 70 types of mutations and variations in the *ETFDH* gene have been reported in riboflavin-responsive MADD (RR-MADD) patients [1,6,7]. In particular, a high prevalence of the c.250G>A (p.Ala84Thr) mutation has been reported in Taiwanese patients with RR-MADD [9].

**Figure 1:**
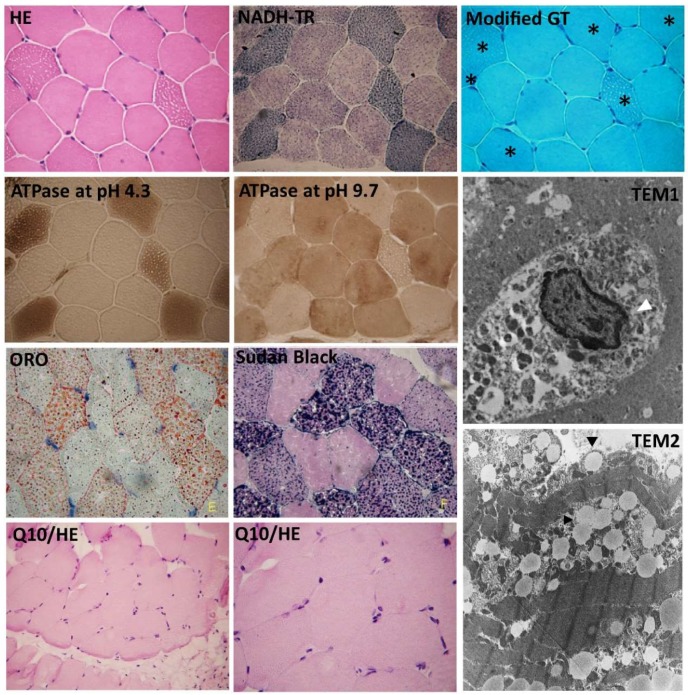
Histological and histochemical findings in muscle biopsies from the MADD patient 1. From left to right: muscle-specific staining with hematoxylin and eosin (HE) stain for myofibril morphology; Nicotinamide adenine dinucleotide (NADH)-tetrazolium reductase (NADH-TR) stain for respiratory complex I enzyme activity and the intermyofibrillar network; Modified Gomori Trichrome stain for demonstrating the intermyofibrillar network and detecting ragged fibers in mitochondrial myopathy; ATPase at pH 4.3, ATPase at pH 9.7 for differentiating type 1 and type 2 myofibers; Oil red O (ORO) for neutral lipids, and Sudan Black for neutral triglycerides and lipids. Stars indicate the affected muscle fibers with vacuolar myopathy in the serial muscle sections. Histochemical staining showed vacuolar myopathy and lipid droplet accumulation in type I muscle sections from MADD patient 1. Transmission electron microscopy (TEM1 and TEM2) images of the muscle ultrastructure are shown. White arrowhead indicates necrotic nucleus; black arrowheads indicate lipid droplets in the sarcolemma of MADD patient 1. Coenzyme Q10 (Q10) therapy has been shown to attenuate vacuolar myopathy in the Q10/HE muscle section.

In the present study, we identified homozygous double mutations, c.250G>A (p.Ala84Thr) and c.92C>T (p.Thr31Ile), that occurred in the MADD family (Figure 1). To date, how the c.250G>A mutation (p.Ala84Thr) and/or c.92C>T (p.Thr31Ile) induces molecular abnormalities into the mitochondrial metabolism has not been well documented. In the present study, we tested whether the genetic variants (c.250G>A and/or c.92C>T) of the *ETFDH* gene elicit a cycle between mitochondrial dysfunction and lipid droplet accumulation and to further investigate the correlation between genotype and phenotype.

## 2. Materials and Methods

### 2.1. Patients

Two male MADD patients were included. Patient 1 (P1) was a 13 year-old Taiwanese adolescent without a familial history of metabolic disease. Patient 1 had tachycardia, facial soreness when he ate and chewed, proximal muscle weakness, and a serum creatine kinase (CK) level of 588 IU/L was noted. A muscle biopsy revealed lipid droplet storage in the skeletal myofibrils, especially in type 1 fibers. After L-carnitine treatment, his CK levels increased further to 45,899 IU/L. His symptoms were relieved after the addition of oral coenzyme Q10 (100 mg/day), and his CK levels returned to 57 IU/L after 2 months. Patient 2 (P2) is the younger brother of P1 and was diagnosed when he was 17 years old. He would get tired after walking 10–20 m and had difficulty standing up from a sitting position. A CK level of 504 IU/L was noted at diagnosis. A muscle biopsy showed lipid storage myopathy. Unfortunately, he had one episode of rhabdomyolysis induced by septic fever and died after a month, even with early supplementation with L-carnitine, coenzyme Q10 and riboflavin.

### 2.2. Mutation Screening

Two male MADD patients, one relative from the affected pedigree and one normal control from an unrelated pedigree were included. This study was performed according to the tenets of the Declaration of Helsinki for research involving human subjects. The protocol was approved by the Ministry of Science and Technology of Taiwan and the Taipei Medical University-Joint Institutional Review Board (TMU-JIRB-N201506002). Whole blood (15 mL) from the study participants was drawn and collected in EDTA-containing tubes. Genomic DNA was isolated from the blood cells using a DNA purification kit (QIAamp DNA Mini kit, Qiagen, Valencia, CA, USA). Primer pairs covering 13 coding exons and the flanking intron splice sites were prepared and used to amplify DNA segments by polymerase chain reaction (PCR) using a DNA thermal cycler (Applied Biosystems GeneAmp PCR system 9700, Thermo Fisher Scientific, Foster City, CA, USA). The PCR products were purified and mixed with a dye terminator cycle sequencing kit (Applied Biosystems) and sequenced using an auto sequencer (Applied Biosystems 3730XL DNA Analyzer, Thermo Fisher Scientific). The putative mutations were tested for segregation in the family by direct sequencing.

### 2.3. Analysis of Blood Acyl-Carnitine Profiles

Saturated (C6-C24 fatty acids, straight-chain kit) and unsaturated (fatty acids unsaturated kit) fatty acid standards were purchased from Sigma–Aldrich (St. Louis, MO, USA). Methanol, acetonitrile and isopropanol were supplied by Burdick & Jackson (Muskegon, MI, USA). The total fatty acids and free fatty acids were extracted and quantified by negative ion chemical ionization gas chromatography coupled with mass spectrometry (GC-MS). A set of deuterated fatty acids was added to the samples to serve as an internal standard. Methanol, hydrochloric acid and deuterated fatty acids were added to samples that were then extracted with iso-octane and derivatized to pentafluorobenzyl esters for GC analysis. The ratios of unlabeled to labeled standard were measured and used to determine the unlabeled analyte levels for samples [10].

### 2.4. Histological and Histochemical Examination

Open muscle biopsies in both patients were done using the left vastus lateralis. The biopsy tissue was divided into three. One part of the tissue was snap-frozen in optimal cutting temperature (OCT) compound in liquid nitrogen for cryosections, the second part was fixed in 3% cacodylate-buffered glutaraldehyde for transmission electron microscopy (TEM), and the third part was fixed in 10% buffered formalin for paraffin-embedded sections. Sections were cut to a thickness of 10 μm on a cryostat at −25 °C. Serial cross-sections of muscle biopsy specimens were obtained for histochemical staining according to standard procedures. The cryosections were stained with hematoxylin and eosin (HE), NADH-tetrazolium reductase stain (NADH-TR), a modified Gomori trichrome stain, Oil red O stain, and Sudan Black stain. Enzymatic histochemistry with adenosine triphosphatase (ATPase) at pH 9.7 and pH 4.3 were done to identify myofibril types.

### 2.5. Transmission Electron Microscope Examination

Muscle fibers were post-fixed in 1% osmium tetroxide for 1 hour. Samples were dehydrated in a graded series of acetone (25%, 50%, 75%, and 100%) before being embedded in epoxy resin (TAAB medium grade) and polymerized at 60 °C. Muscle tissue sections (70-nm thick) were then transferred to copper grids, stained with uranyl acetate and lead citrate, and examined with a Hitachi H-600 electron microscope (Nissei Sangyo, Tokyo, Japan). The ultrastructural appearance of myofibrils was analyzed and imaged at a 7900× magnification.

### 2.6. Cell Culture

We established four lymphoblastoid cell lines from P1, P2, a normal control (wild type, WT), and the Family I-1 (carrier 1, C1). Epstein-Barr virus-transformed lymphoblastoid cell lines from all participants were generated by the Food Industry Research and Development Institute (BCRC, Hsinchu, Taiwan). The cell lines were maintained and grown at 37 °C and a humidified 5% CO_2_ in RPMI 1640 medium supplemented with 15% fetal calf serum (FCS, GIBCO, Grand Island, NY, USA), 1 mM pyruvate, 50 μg/mL uridine, 100 unit/mL of penicillin, and 0.1 mg/mL of streptomycin. The lymphoblastoid cells were challenged with palmitic (C16:0), capric (C10:0) or hexanoic (C6:0) acid. The concentrations of fatty acids used varied from 0.1 to 1.0 mM Palmitic acid was conjugated to fatty acid-free bovine serum albumin (BSA). The cells were cultured with the BSA-conjugated palmitic, capric, or hexanoic acid at 37 °C and 5% CO_2_ for 24 h [11].

### 2.7. Reverse-Transcription and Real-Time Quantitative PCR for ETFDH Expression

Total RNA was extracted with an RNeasy Mini Kit (Qiagen, Valencia, CA, USA). First-strand cDNA synthesis was performed with 5 U of MMLV reverse transcriptase (Epicentre, Madison, WI, USA), 1 μg of RNA and 50 pmol of primers (Promega, Madison, WI, USA). Real-time quantitative PCR (qPCR) was performed using a 48-well, StepOne™ real-time PCR instrument (Applied Biosystems, Foster City, CA, USA). The reactions were carried out in triplicate. The threshold cycle numbers for β-actin (*ACTB*) and the *ETFDH* gene were measured. The following qPCR primers were used: *ETFDH* forward: 5′-GCAGCTGCTGAGGTCCTTTT3′, *ETFDH* reverse: 5′-TGTCCAAGGGCCAACCAACA-3′, *ACTB* forward: 5′-CCAACCGCGAGAAGATGA-3′, and *ACTB* reverse: 5′-CCAGAGGCGTACAGGGATAG-3′.

### 2.8. Western Blot Analysis for ETFDH Expression

Protein samples (40 μg) were subjected to 10% sodium dodecyl sulfate polyacrylamide gel electrophoresis and then transferred to a polyvinylidene fluoride or polyvinylidene difluoridemembrane (GE Healthcare Bio-sciences, Fribourg, Switzerland). Immunoblotting was performed with an anti-ETFDH (1:1000, SC-242642, Santa Cruz Biotechnology, Santa Cruz, CA, USA) or anti-β-actin (1:2000, GTX-110564, GeneTex, San Antonio, TX, USA), primary antibody, a horseradish peroxidase-conjugated anti-mouse immunoglobulin G secondary antibody (#58802, Cell Signaling Technologies, Beverly, MA, USA), and enhanced chemiluminescence detection was completed by a LAS 4000 chemiluminescent imager and a GFP CCD Imager (ECL, GE Healthcare Bio-Sciences).

### 2.9. ATP Assay

Cellular ATP levels were determined by luciferin- and luciferase-based assays. cells were lysed with ATP-releasing buffer and quantified using an ATP assay kit (Perkin Elmer Inc., Waltham, MA, USA). The supernatant was detected on a Wallac Victor 1420 Multi-label Counter (Perkin Elmer Inc.).

### 2.10. Flow Cytometry Analysis of Mitochondrial Membrane Potential (ΔΨm) and Lipid Droplet and Lipid Peroxide Levels

Aliquots of 1 × 10^6^ cells were gently stained in the dark for 15 minutes at 25 °C with either 5 µM JC-1 (for mitochondrial membrane potential) (Molecular Probes, Thermo Fisher Scientific, Madison, WI, USA), Nile red (for neutral lipid droplets, Molecular Probes), or C11-BODIPY®^581/591^ C11 (for lipid peroxides, Molecular Probes). After staining, all analyses were performed with a FACScan (Becton Dickinson, San Jose, CA, USA) equipped with a 100 mW Argon laser. A minimum of 30,000 cells per sample were analyzed. Debris was gated out based on light-scatter measurements. The data were quantified using the CellQuest software (version 3.1) (Becton Dickinson).

### 2.11. Seahorse XF24 Metabolic Flux Analysis for Mitochondrial Respiration

To investigate the respiratory capacity of the cells, intact cellular respiration was detected by the Seahorse XF24 Metabolic Flux assay (Agilent Seahorse Bioscience, Chicopee, MA, USA). Cells cultured in XF24-well micro plates at 4 × 10^4^ cells/well and incubated at 37 °C and 5% CO_2_ were treated for 24 h with fatty acids. Baseline measurements were recorded before the addition of 3 μM oligomycin (an inhibitor of respiratory complex V), 1 μM carbonylcyanide m-chlorophenylhydrazone (CCCP, a mitochondrial uncoupler) and 5 μM rotenone (an inhibitor of respiratory complex I). The oxygen consumption rate (OCR) was automatically calculated and recorded by Seahorse XF24 software. The protein concentration of each well was determined with a Pierce bicinchoninic acid assay (Thermo Fisher Scientific, Rockford, IL, USA).

### 2.12. Oil Red O and Nile Red Staining for Lipid Droplets

#### 2.12.1. Oil Red O Staining

A stock Oil Red O solution was prepared by dissolving 300 mg of Oil Red O powder in 100 mL of 99% isopropanol. Fresh working solution was prepared by diluting the Oil Red O stock solution in distilled water in a 3:2 volume ratio and filtering before use. Aliquots of 1 × 10^6^ cells were treated with 400 μM palmitic acid (C16:0) or a combination of palmitic acid, 540 nM riboflavin, and/or 100 μM coenzyme Q10 for 24 h. The cells were fixed with 10% formalin and treated with Oil Red O working solution for 5 min at 25 °C. Hematoxylin was added to the slide and incubated for 1 min at 25 °C, then removed and the slide rinsed with running tap water. The oil droplets were observed under a phase contrast microscope, lipids appeared red and nucleoli appeared blue.

#### 2.12.2. Nile Red Staining

A stock solution of Nile Red (Sigma N-3013) in acetone (250 mg/mL) was prepared. A working solution was prepared by diluting the Nile Red stock solution with a 50 mM Tris/maleate and 2–3% *w*/*v* polyvinylpyrrolidone solution in a 1:100 ratio (*v*/*v*). Aliquots of 1 × 10^6^ cells were stained with 100 μL drops of Nile Red for 5 min at 25 °C. The stained cells were viewed under a Nikon microscope with an EFD-3 episcopic fluorescence attachment and a B-2A filter (excitation at 450–490 nm, 505 nm dichroic mirror, and 520 nm barrier filter).

### 2.13. Cloning and Transfection of ETFDH Variants

To distinguish the role of the ETF-QO variants on lipid droplet accumulation, we further established specific lymphoblastoid cells that differentially overexpressed the c.92C>T, c.250G>A, or coexisted c.92C>T and c.250G>A (c.92C>T + c.250G>A) *ETFDH* mutations. We cloned and extracted five types of plasmids, including pEGFP-C1, pcDNA3.1-*ETFDH* WT, pcDNA3.1-*ETFDH* c.92C>T (T31I), pcDNA3.1-*ETFDH* c.250 G>A (A84T), and pcDNA3.1-*ETFDH* c.92C>T combined with c.250G>A (p.T31I/p.A84T), using a Geneaid Plasmid Maxi Kit (Geneaid Biotech, New Taipei, Taiwan). We further transfected the control lymphoblastoid cells with one of the five plasmids using X-tremeGENE HP DNA transfection reagent (Roche Diagnostics GmbH, Mannheim, Germany). Approximately, 1 μg/100 μL plasmids and 2 μL X-tremeGENE HP DNA transfection reagent were mixed together. The transfection mixtures were allowed to incubate in the tissue culture flasks at 37 °C for 10 min before the control lymphoblastoid cells were added. Cells were transfected at 37 °C in 5% CO_2_ for 18 h.

### 2.14. Statistical Analysis

Paired T-tests and one-way ANOVA were used for data analysis. A *P* value < 0.05 was considered significant. Plots show the mean ± standard deviation (S.D.).

## 3. Results

### 3.1. Abnormal Profiles of Plasma Fatty Acid Composition and Metabolic Parameters in MADD Patients

One asymptomatic relative (Carrier 1, C1) and two affected, multiple acyl-CoA dehydrogenase deficiency (MADD) patients (P1 and P2) were enrolled in the study. The plasma fatty acid and metabolic profiles of the study participants are shown in Table 1. Both MADD patients showed abnormal acylcarnitine profiles when compared with the normal range. The bold values indicate abnormal levels of free carnitine and acyl carnitine. Acyl-carnitine profiles of short-, medium- and long-chain fatty acid metabolism were elevated.

### 3.2. Increased Lipid Droplet Accumulation in the Sarcolemma in MADD Patient 1

Muscle-specific staining was performed on the cryosection of muscle tissue from P1 (Figure 1). All staining of muscle tissue was performed on serial sections. Modified Gomori Trichrome staining represented the compensation response for mitochondrial function deficiency. Red droplets from Oil Red O (ORO) staining indicate the intracellular accumulation of lipid droplets. Sudan black B was used to identify lipids, including neutral fats, phospholipids, and sterols, in the muscle tissue sections. Together with ATPase at pH4.3 and pH9.7 staining, lipid storage was identified in type I muscle fibers. The muscular ultrastructure was observed by Transmission Electron Microscopy (TEM). Nuclear condensation and abnormal mitochondrial structure were found in TEM1 and TEM2 sections, respectively. Lipid droplets were accumulated in the sarcolemma.

### 3.3. The Identification of the c.92C>T and c.250G>A Mutations in the Affected MADD Patients

**Figure 2:**
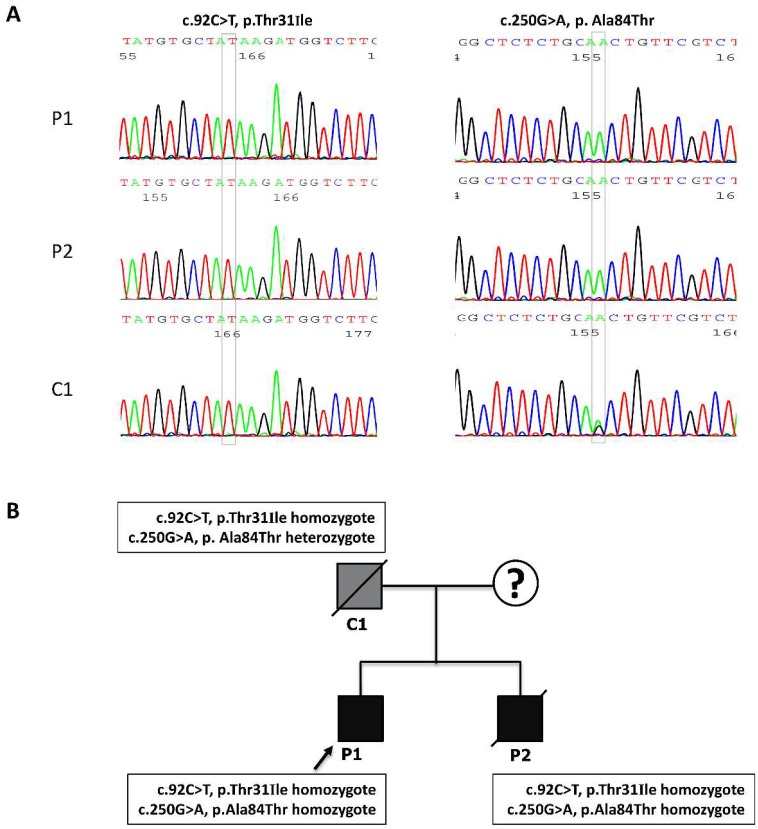
A genetic chromatogram of the electron transfer flavoprotein dehydrogenase (*ETFDH*) gene and genotypes of the MADD family members. (**A**) A sequence analysis of the *ETFDH* gene was performed. Direct sequencing revealed a c.92C>T polymorphism in exon 2 that resulted in a p.Thr31ILe homozygote mutation in the father 1 (C1), Patient 1 (P1), and Patient 2 (P2). Another mutation, a c.250G>A transition mutation in exon 3, resulted in a p.Ala84Thr homozygous mutation, which was found in P1 and P2. A c.250G>A heterozygous mutation was found in the father. (**B**) A pedigree diagram showing the proband (arrow) and the relatives of the MADD family. The mother was unavailable. Four lymphoblastoid cell lines were established from the unrelated normal control (wild type, WT), the father with a c.250G>A *ETFDH* heterozygous mutation and a c.92C>T homozygote mutation (C1), and two affected patients who both had c.92C>T and c.250G>A homozygous mutations.

We performed a mutation analysis in C1, P1, and P2 of the MADD family. The direct sequencing revealed two types of homozygous *ETFDH* mutations in P1 and P2: a c.92C>T mutation in exon 2 resulting in a p.Thr31ILe mutation and a c.250G>A mutation in exon 3 resulting in a p.Ala84Thr mutation. A homozygous c.92C>T mutation and heterozygous c.250G>A mutation was found in C1 (Figure 2). No mutations in mitochondrial DNA were detected in the MADD family.

### 3.4. The Reduced Expression Levels of ETFDH mRNA and ETF: QO in the ETFDH-Mutated Lymphoblastoid Cells

**Figure 3:**
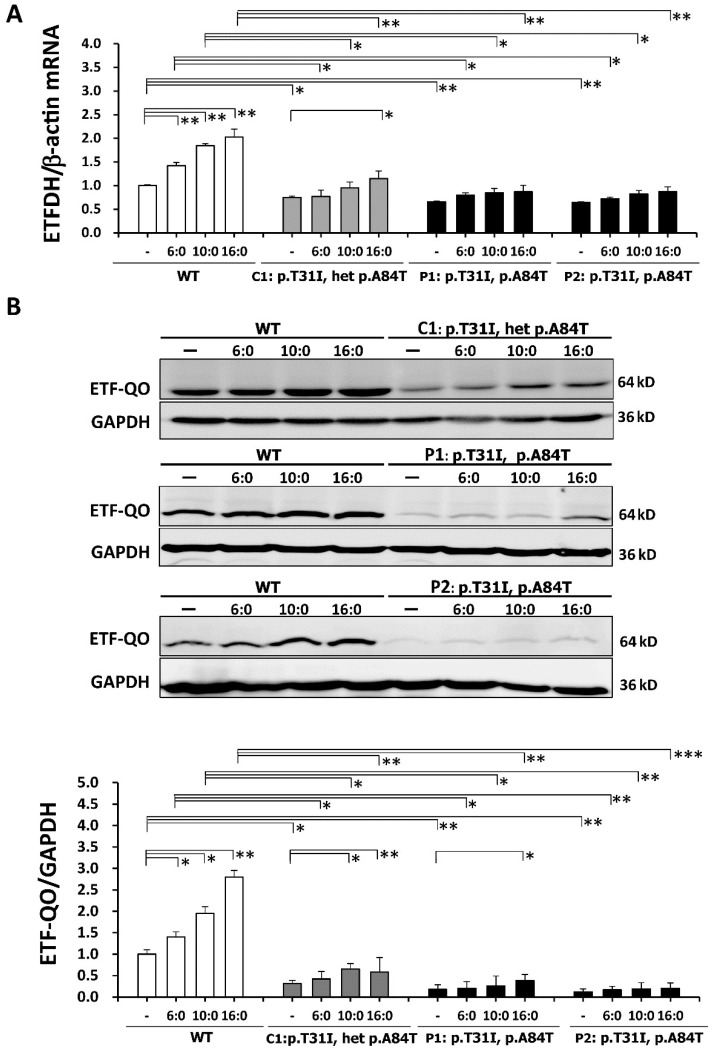
*ETFDH* mRNA and the electron-transfer flavoprotein ubiquinone oxidoreductase (ETF-QO) protein expression profiles in response to treatment with different types of fatty acids. The four types of lymphoblastoid cells were treated with 400 μM of palmitic (C16:0), capric (C10:0) or hexanoic (C6:0) acid for 18 hours. (**A**) ETFDH mRNA levels were detected in the cultured lymphoblastoid cells by quantitative real-time polymerase chain reaction (PCR). Reduced mRNA levels were found in C1, P1, and P2 cells. (**B**) ETF-QO protein levels, as measured by western blot, were detected in cultured lymphoblastoid cells. Equal loading was assessed by glyceraldehyde 3-phosphate dehydrogenase (GAPDH) (lower panel). MADD P1 and P2 showed significantly decreased levels of ETF-QO protein and an impaired response to fatty acid treatment compared with the normal control (wild type, WT). The plots are presented as the mean ± standard deviation (SD) of four independent experiments. Statistical significance: *, *P* < 0.05; **, *P* < 0.01; ***, *P* < 0.001.

The four lymphoblastoid cell lines (normal control (WT), C1, P1, and P2) were treated with 400 μM of palmitic (C16:0), capric (C10:0), or hexanoic (C6:0) acid for 18 hours. In response to treatments with fatty acids, the expression levels of *ETFDH* mRNA were significantly increased (0.8-fold) in WT cells compared with untreated WT cells (Figure 3A). Lower expression levels of *ETFDH* mRNA were found in P1 and P2 cells with or without various fatty acid treatments (Figure 3A). We performed western blotting to detect the ETF-QO protein levels in the carrier and affected patients. A band size of 64 kDa was observed. Remarkable 1.7-fold and 2.6-fold increases of ETF-QO protein levels were detected in the capric acid- and palmitic acid-treated WT cells, respectively, compared to untreated WT cells. P1 and P2 cells showed a significant decrease in ETF-QO protein with or without various fatty acid treatments compared to WT cells (Figure 3B).

### 3.5. ETFDH Mutations Caused Mitochondrial Dysfunction

**Figure 4:**
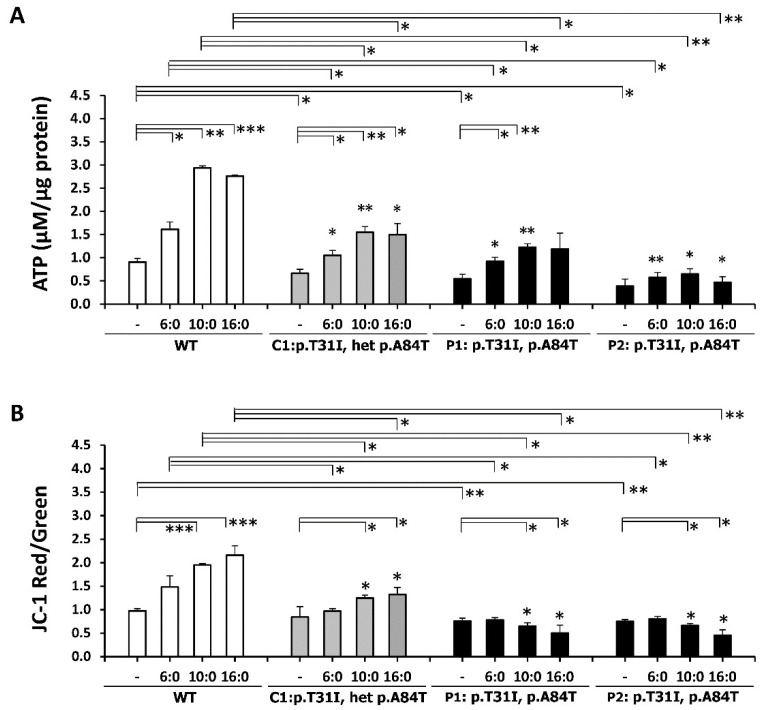
Effect of *ETFDH* variations on mitochondrial function in response to different types of fatty acids. Four lymphoblastoid cell lines were treated with 400 μM of palmitic (C16:0), capric (C10:0) or hexanoic (C6:0) acid for 18 h. (**A**) Adenosine triphosphate (ATP) content was measured by the Luminescence ATP assay system. Significantly declined ATP levels were found in the P1 and P2 cells. (**B**) The mitochondrial membrane potential of the cultured cells was stained with JC-1 and analyzed by flow cytometry. The ratio of JC-1 Red (aggregates)-to-Green (monomer) fluorescence was significantly decreased in P1 and P2 cells. The adverse effects of fatty acid treatment on the mitochondrial membrane potential in P1 and P2 cells were different than those in WT and C1 cells. The plots are presented as the mean ± SD (*n* = 4). Statistical significance: *, *P* < 0.05; **, *P* < 0.01; ***, *P* < 0.001.

The lymphoblastoid cells were treated with 400 μM of palmitic (C16:0), capric (C10:0), or hexanoic (C6:0) acid for 18 hours. Compared with untreated WT cells, significant decreases of ATP content and mitochondrial membrane potential were detected in P1 and P2 cells. Nevertheless, ATP synthesis responded to fatty acid treatment in all four cell lines (Figure 4A). In addition, the ratio of JC-1 aggregate/monomer showed 0.84-, 0.75-, and 0.73-fold decreases in the control group in C1, P1, and P2 cells, respectively (Figure 4B). Different to the positively responsive WT and C1 cells, the P1 and P2 cells had a negative mitochondrial membrane potential response to fatty acid treatment. The profound detrimental effects of palmitic acid treatment on mitochondrial function were shown in the MADD cells.

### 3.6. ETF-QO Is Involved in the Maintenance of Mitochondrial Bioenergetics and Respiratory Coupling

**Figure 5:**
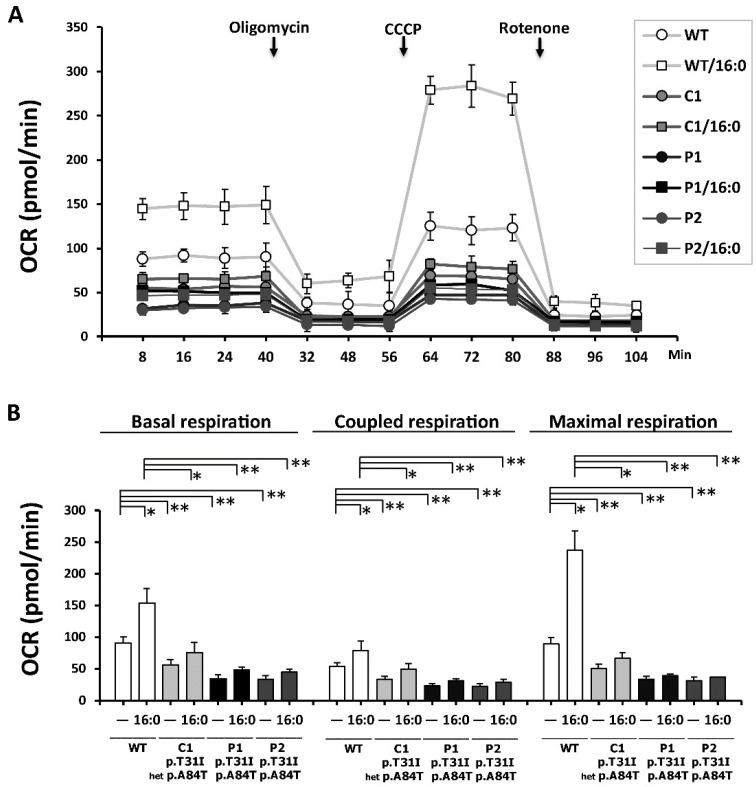
ETF-QO involved in the maintenance of mitochondrial bioenergetics and coupling of mitochondrial respiration. Mitochondrial respiration was assessed using the Seahorse XF24 Metabolic Flux Analyzer. (**A**) Data are presented as time courses of the oxygen consumption rate (OCR) under basal conditions, followed by the sequential addition of oligomycin (1 μg/mL), carbonylcyanide m-chlorophenylhydrazone (CCCP) (1 μM), and rotenone (10 μM). (**B**) The progress curve is annotated to show the relative contribution of basal respiration, coupled respiration (after the addition of oligomycin), and maximal respiration (after the addition of CCCP and rotenone). Basal, coupled, and maximal oxygen consumption rates were assessed in the presence or absence of 400 μM of palmitic acid (C16:0) for 18 h. C1, P1, and P2 cells showed significant decreases in basal respiration, coupled respiration, and maximal respiration. The plots are presented as the mean ± SD (*n* = 4). Statistical significance: *, *P* < 0.05; **, *P* < 0.01.

In Figure 5, mitochondrial respiration and bioenergetics is represented as the time course change of the OCR under basal conditions followed by the sequential addition of oligomycin (3 μg/mL), carbonylcyanide m-chlorophenylhydrazone (CCCP) (1 μM), and rotenone (3 μM). The progress curve is annotated to show the relative contribution of basal respiration, coupled mitochondrial respiration (OCR change between the basal condition and the addition of oligomycin) and maximal respiration (OCR change between the addition of CCCP and rotenone). The time-dependent OCR progress curves of WT, C1, P1, and P2 cells are shown in Figure 5A. Significantly lower basal respiration, coupled respiration (ATP-related respiration), and maximal respiration were found in C1, P1, and P2 cells compared with WT cells. A remarkable increase in maximal respiration was found in WT cells treated with palmitic acid. However, only a slight increase in basal, coupled, and maximal respiration occurred in the C1, P1, and P2 cells treated with palmitic acid (Figure 5B).

### 3.7. Riboflavin and Coenzyme Q10 Reduced the Intracellular Accumulation of Neutral Lipid Droplets and Lipid Peroxides in ETFDH-Mutated Cells

**Figure 6:**
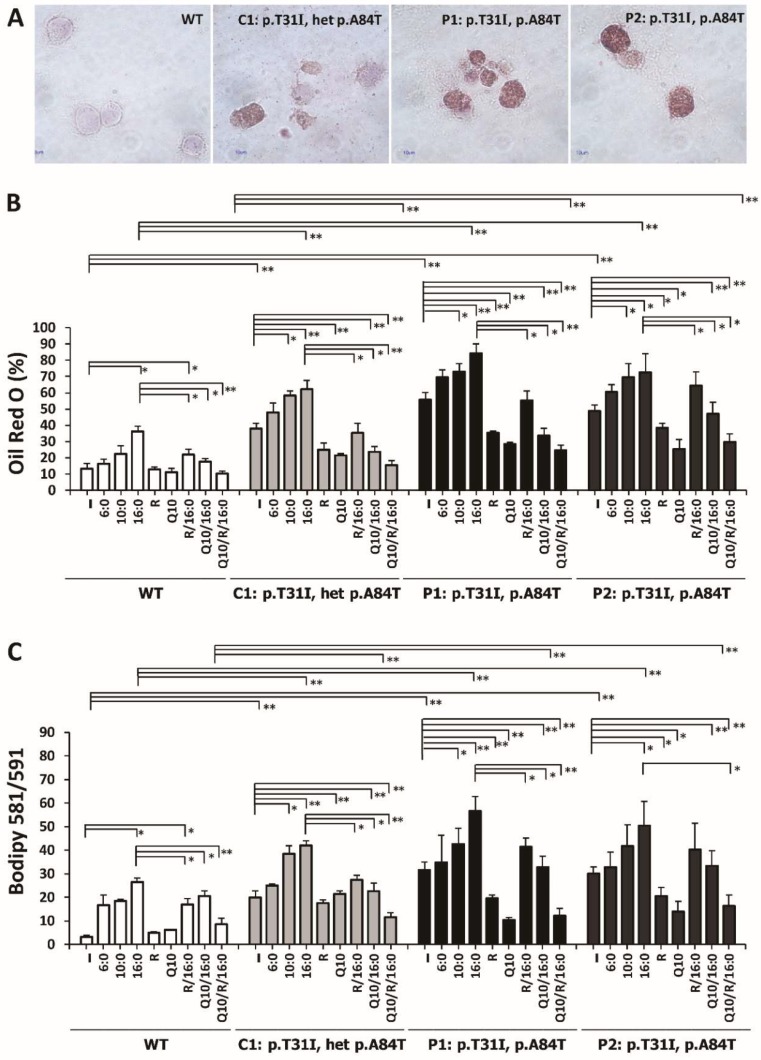
Effect of riboflavin and coenzyme Q10 on the intracellular accumulation of lipid droplets and lipid peroxides. The lymphoblastoid cells were treated with 400 μM of palmitic acid (C16:0) or a combined treatment of palmitic acid with 540 nM riboflavin and/or 100 μM coenzyme Q10 for 18 hours. (**A**) The images of Oil Red O-positive cells in WT, C1, P1, and P2 cells are shown. (**B**) The percentage of Oil Red O-positive cells in P1 and P2 cells after 540 nM riboflavin and/or 100 μM coenzyme Q10 treatments were analyzed. (**C**) The mean fluorescent intensity of BODIPY®^581/591^. The levels of lipid peroxide were detected by BODIPY ®^581/591^ staining and flow cytometry analysis. The increased levels of neutral lipid droplets and lipid peroxides were demonstrated in MADD cells. A remarkable decrease in lipid droplet accumulation occurred with the combined treatment of palmitic acid with riboflavin and coenzyme Q10. The plots are presented as the mean ± SD (*n* = 4). Statistical significance: *, *P* < 0.05; **, *P* < 0.01.

Images of ORO staining are shown in Figure 6A. Intensive ORO-staining occurred in P1 and P2 cells. The percentages of ORO-positive WT, C1, P1, and P2 cells after different treatment types (different fatty acids with/without 540 nM riboflavin for 18 hours) were compared. There were approximately 13.5%, 38.3%, 55.7% and 48.6% ORO-positive fatty acid un-treated WT, C1, P1, and P2 cells, respectively. After palmitic acid treatment, there were approximate 3.4-, 4.6-, 6.2 and 5.4-fold and increases in ORO-positive cells in the palmitic acid-treated WT, C1, P1, and P2 cells, respectively. Supplementation of riboflavin decreased the percentage of ORO-positive palmitic acid-treated patient-derived cells compared with untreated patient-derived cells (Figure 6B). In addition, the most effective improvement in fatty acid metabolism was shown in riboflavin and coenzyme Q10 co-supplemented cells (Figure 6B). Lipid peroxide levels were detected by BODIPY®^581/591^ C11 staining and flow cytometry analysis. Compared with WT cells, there were approximate 6.2-, 9.8-, and 9.4-fold increases in lipid peroxides in C1, P1, and P2 cells, respectively. In WT cells treated with hexanoic acid, capric acid, palmitic acid, or a combination of riboflavin and palmitic acid, there were increases in lipid peroxide levels of approximately 5.2-, 5.7-, 8.2-, and 5.3-fold, respectively. Riboflavin, co enzyme Q10, and Riboflavin/coenzyme Q10 combined treatment significantly reduced the accumulation of lipid peroxides in the palmitic acid-treated P1 and P2 cells (Figure 6C).

### 3.8. Both the c.250G>A and c.92C>T ETFDH Variants Induced the Intracellular Accumulation of Neutral Lipid Droplets

**Figure 7:**
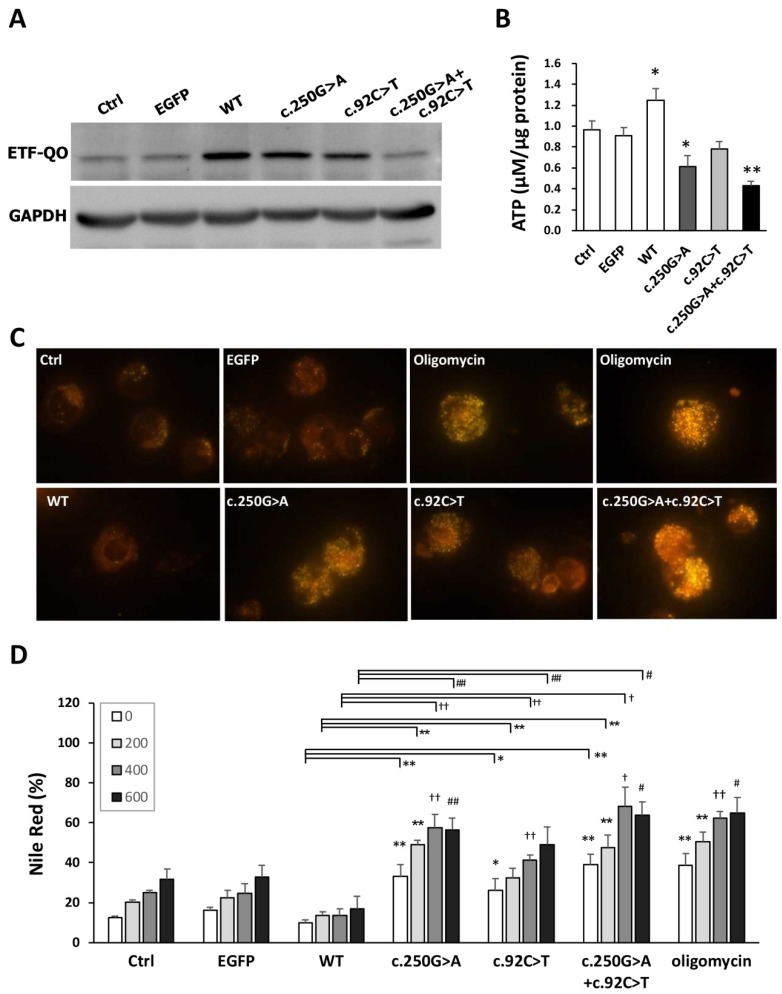
Differential effect of *ETFDH* wild type and variations on the accumulation of intracellular lipid droplets. Lymphoblastoid cells were transfected with five types of plasmids: pEGFPC1, pcDNA3.1-wild type ETFDH (WT), pcDNA3.1-c.92C>T (T31I), pcDNA3.1-c.250 G>A (A84T), and pcDNA3.1-c.250G>A + c.92C>T (p.T31I + p.A84T). The oligomycin group was treated with 1.5 μM oligomycin (the respiratory chain inhibitor). (**A**) Representative immunoblot of ETF-QO protein was shown. (**B**) The ATP levels were measured by the Luminescence ATP assay system. Significantly declined ATP levels were found in the c.250 G>A and c.250G>A + c.92C>T mutant cells. (**C**) The images of Nile Red stained cells were shown. All cells were treated with or without 400 μM palmitic acid (C16:0) for 18 hours and stained with 0.5 μg/mL Nile Red for 30 minutes. (**D**) The percentages of Nile Red positive cells were shown. The cells were differentially treated with 200 μM, 400 μM, and 600 μM of palmitic acid, and the percentages of Nile Red positive were compared. The plots are presented as the mean ± SD (*n* = 4). Ctrl, the normal control lymphoblast cells without plasmid transection, EGFP, enhanced green fluorescent protein. Statistical significance: *, *P* < 0.05 and **, *P* < 0.01 for comparison to the control cells without palmitic acid treatment or intergroups; *, *P* < 0.05 and **, *P* < 0.01 for comparison to the control cells with 200 μM palmitic acid treatment or intergroups; ^†^, *P* < 0.05 and ^††^, *P* < 0.01 for comparison to the control cells with 400 μM palmitic acid treatment or intergroups; ^#^, *P* < 0.05 and ^##^, *P* < 0.01 for comparison to the control cells with 600 μM palmitic acid or intergroups.

To clarify the contributions of c.250G>A and c. 92C>T *ETFDH* variations to MADD, the three types of *ETFDH* mutations (c.250G>A, c.92C>T, and coexisted c.250G>A and c.92C>T (c.250G>A + c. 92C>T) were cloned from P1 cells. We constructed four different clones that harbored EGFP (vector control, Ctrl), wild type (WT), c.92C>T (p.T31I), c.250G>A (p.A84T), or c.92C>T + c.250G>A (p.T31I + p.A84T) and transfected these plasmids into normal control cells. The representative western blots for the ETF-QO protein levels of the mutants in these cell lines were shown in Figure 7A. The significantly declined ATP levels were found in the c.250 G>A and c.250G>A + c.92C>T transfected cells in Figure 7B. In addition, the transfected lymphoblastoid cells were treated with 400 μM of palmitic acid for 18 h and stained with Nile Red. The images of Nile Red staining are shown in Figure 7C. The percentages of Nile Red-positive WT, c.92C>T, c.250G>A, and c.92C>T + c.250G>A cells treated with 200 μM, 400 μM, and 600 μM of palmitic acid were compared. Without fatty acid treatment, there were approximately 12.33%, 16.3%, 10%, 33%, 26%, 39%, and 38.6% Nile Red-positive Ctrl, EGFP, WT, c.250G>A, c.92C>T, c.92C>T + c.250G>A, and oligomycin-treated cells, respectively (Figure 7D). Compared with EGFP cells, there were approximate 0.22-, 1.8-, 1.3-, and 2.7-fold increases in Nile Red-positive cells in the 400 μM palmitic acid-treated normal control, WT, c.250G>A, c.92C>T, and 92C>T + c.250G>A cells, respectively. The dose-responsive accumulation of lipid droplets was found in the mutant cells. The overexpression of wild type ETF-QO significantly reduced the palmitic acid-induced lipid droplet accumulation. Cells with the c.92C>T + c.250G>A plasmid transfection had the highest lipid droplet accumulation (Figure 7D), and were vulnerable to cell death in 600 μM palmitic acid treatment (data not shown). However, there was no significantly difference in the ATP levels and lipid droplet contents between c.250G>A and c.250G>A + c.92C>T. In addition, increased Nile Red-positive cells were observed in the oligomycin treatment indicating the impaired respiratory chain result in lowered capacity for fatty acid β-oxidation in mitochondria.

## 4. Discussion

MADD is a fatty acid oxidation disorder pathologically characterized by high levels of acyl-carnitines in tissues and body fluids and the accumulation of lipid droplets in type I muscle fibers of affected individuals. The genetic data of 440 late-onset MADD patients was reviewed and the majority of causative mutations were found in *ETFDH* genes [1]. Most patients with late-onset MADD, reported in Taiwan and southern China [12,13], carried a homozygous mutation of c.250G>A in *ETFDH* [11,12]. In the present study, two late-onset affected patients with ETF-QO deficiency were shown to harbor both c.92C>T and c.250A>G homozygous mutations using sequence analysis. Here, we verified and confirmed all three types of the c.92C>T, c.250A>G, and c.92C>T + c.250A>G mutations that lead to the pathological accumulation of lipid droplets. To the best of our knowledge, our study is the first demonstration that overexpression of the human c.250G>A and/or c.92C>T *ETFDH* mutants contributes to dysfunctional fatty acid oxidation and mitochondrial metabolism.

The *ETFDH* c.250G>A gene mutation in exon 3 is a hotspot mutation that harbors a high allelic frequency of 0.004 and a high carrier frequency of 0.8% (1:125) in the Taiwanese population [12,14,15]. Based on the prediction of its three-dimensional (3D) structure and simulations of its molecular dynamics, Er et al. reported that the *ETFDH* c.250G>A mutation is located within the FAD binding domain and is pathogenic for altering the ETF-QO protein structure near the FAD binding site as well as disrupting the site’s binding stability with FAD [13]. The c.92T>C mutation (T31I, rs11559290) in exon 2 of the *ETFDH* gene is located within the mitochondrial targeting peptide domain and is considered to be neutral due to its uncharged residue in the leader sequence of the peptide. Meanwhile, the allele frequency of T31I in a population in the USA was much more prevalent, with a frequency of 0.27 [16]; furthermore, it has been determined to be 0.13 in a Danish population [4], but only 0.027 in a Chinese population [17].

In addition to lipid droplet deposition in muscle tissue, MADD patients also have lipid vacuoles that correspond to Jordan’s anomaly in leukocytes [18,19]. Experimental evidence demonstrated that using lymphocytes instead of fibroblasts for diagnosis of fatty acid oxidation disorders to evaluate the efficiency of palmitate β-oxidation in human leukocytes can serve as a simple and rapid assay to assess mitochondrial metabolic capacity [20,21,22]. In addition, studies have shown that the fatty acids act as intracellular and intercellular mediators in lymphocytes to regulate DNA synthesis, cytokine production, cell growth, and immune responses [23,24,25]. These data support the use of lymphoblastoid cell lines from affected individuals as cell models for functional assessments. We confirmed that the use of lymphoblastoid models for the study of *ETFDH* mutations in mitochondrial metabolism and lipid droplet formation in MADD skeletal muscle was reasonable.

The acylcarnitine profile of MADD patient 1 by tandem mass spectrometry showed abnormalities in the short-, medium-, and long-chain fatty acid metabolisms. We tested the responses of ETF-QO variants to treatments with palmitic (C16:0), capric (C10:0), and hexanoic (C6:0) acids. *ETFDH* mRNA and protein expression increased in response to fatty acid treatment in the WT cells, and the highest expression levels were found in the palmitic acid treatment group. However, the MADD cells responded poorly to the fatty acid treatments. Additionally, MADD P1 and P2 cells had barely detectable ETF-QO protein levels after fatty acid treatment. To determine whether the low protein level was due to protein instability, we conducted a cycloheximide chase assay and found that ETF-QO variants displayed protein instability (data not shown). A similar finding was found by Olsen et al., where the c.158A>G *ETFDH* missense variation in exon 2 induced ETF-QO protein degradation in patient samples [26]. The causes of ETF-QO protein lability in RR-MADD are probably due to structural abnormalities, folding defects, and thermal instability [27,28]. However, these causes do not rule out the *ETFDH* gene mutation resulting in the dominant negative forms of ETF-QO. According to Olsen’s report, the profound deficiency of ETF-QO activities (1% of the controls mean) was detected in skeletal muscle mitochondria from MADD patients with p.Ala12fs and p.Gly429Arg ETF-QO mutants.

To determine the effect of *ETFDH* mutations on the bioenergetics capacity of mitochondria, we assessed and confirmed that there was a significant reduction in ATP production and mitochondrial membrane potential in the MADD cells and the carrier C1 cells. The magnitude of mitochondrial dysfunction was related to an insufficient amount of ETF-QO protein. Based on oxygen consumption analysis, all the MADD cells showed significant decreases in OCR under glucose or palmitic acid treatments. In addition, the MADD cells had a discordant response to increased workloads. Similarly, there was a significant increase in the extracellular acidification rate in MADD patient fibroblasts that resembled increased glycolysis when compared with control fibroblasts [29]. Moreover, the expression profiles of genes in the glycolytic pathway were significantly induced in MADD patient fibroblasts [29]. This supports the fact that *ETFDH* mutations significantly influence mitochondrial bioenergetics, leading to a metabolic shift from oxidative phosphorylation to glycolysis.

Defects in fatty acid metabolism often cause intracellular accumulation of lipid droplets. Our results showed increased accumulation of lipid droplets in the muscle sarcolemma in MADD patient 1 and in MADD cells. A remarkable increase in lipid droplet formation was found in palmitic acid-treated MADD cells and was attenuated by riboflavin supplementation. The beneficial effects may be related to the riboflavin binding enhancing ETF-QO protein folding, assembly, stability, and catalytic activity [4,30,31]. It is worth noting that coenzyme Q10 supplementation also resulted in a significant improvement in palmitic acid metabolism and reduced lipid droplet accumulation. It has been shown that late-onset MADD patients with *ETFDH* mutations frequently have secondary coenzyme Q10 deficiencies [32]. It is also important to note that riboflavin supplementation attenuates *ETFDH* mutation-induced secondary coenzyme Q10 deficiency. Contrary to secondary coenzyme Q10 deficiency in MADD fibroblasts, Wen et al. reported no significant decrease in coenzyme Q10 levels in the muscle tissue of MADD patients; rather, there was an increase in total coenzyme Q10 levels in the muscle tissue of RR-MADD patients with *ETFDH* gene mutations [33]. In our study, we found that coenzyme Q10 supplementation did not affect ETF-QO protein levels (data not shown). Coenzyme Q10 supplementation might effectively reduce lipid droplet accumulation by improving the efficacy of mitochondrial respiration.

Oxidative damage analysis showed increased accumulation of lipid peroxides in MADD cells. The oxidation of palmitoyl carnitine by isolated mitochondria leads to the generation of H_2_O_2_ [34]. RR-MADD patient fibroblasts suffer from mitochondrial dysfunction and oxidative stress [35]. Treatment with coenzyme Q10 may decrease the level of mitochondrial superoxides in patient cells [36]. Our results showed that riboflavin supplementation significantly reduced lipid peroxide generation. However, in a previous study, riboflavin could not fully rescue the defect in mitochondrial superoxide production in cells from RR-MADD patients cells [35]. Lipid droplets are intracellular organelles that regulate lipid metabolism and energy homeostasis [37]. Excess lipid droplet accumulation has been noted to be the source of lipid peroxidation and leads to the formation of secondary reactive species e.g., aldehydes, which promote oxidative cell injury [38]. Hence, we speculate that riboflavin supplementation might reduce lipid peroxidation by improving palmitic acid metabolism, which reduces the accumulation of lipid droplets.

In the carrier-derived C1 cells that harbored the c.250G>A heterozygous and c.92C>T homozygous mutations, there was less than 31% protein expression and a 5.42-fold increase in neutral lipid droplet formation. The C1 cells also had an affected fatty acid metabolism and mitochondrial dysfunction even though the c.92C>T variant has not been considered to be pathogenic [39]. To functionally characterize these mutations, we established *ETFDH*-mutated cells transfected with plasmids containing the c.92C>T, c.250G>A, or c.92C>T + c.250G>A mutation. A significant ATP dissipation and lipid droplet accumulation was found in the transfected ETFDH mutant cells. However, the ATP level cannot be directly referred to the mitochondrial function and the oximetry analysis of each mutant clone should be addressed in the future. According to our model, a significantly increase in Nile Red-positive lipid droplets was revealed in lymphoblasts overexpressing c.92C>T. In addition, the highest amount of Nile Red-positive lipid droplets was detected in the lymphoblasts with c.250G>A + c.92C>T mutations. Therefore, we concluded that the c.92C>T variant, despite previously been noted as a neutral mutation, have pathological effects.

## 5. Conclusions

The c.250G>A and/or c.92C>T mutations in ETF-QO reduced expression levels of *ETFDH* mRNA and ETF-QO protein, decreased ATP synthesis, dissipated mitochondrial membrane potentials, impaired mitochondrial bioenergetics, induced neutral lipid droplet, and lipid peroxide accumulation, and weakened cellular capacity for fatty acid oxidation. Additionally, riboflavin and coenzyme Q10, which link mitochondrial fatty acid β-oxidation to the mitochondrial respiratory chain, attenuated lipid-induced mitochondrial stress in the *ETFDH*-mutated cell lines. The defective ETF-QO elicits a vicious cycle between mitochondrial dysfunction and lipid droplet accumulation. This demonstration of the genetic basis for MADD provides molecular insight into mitochondrial dysfunctions induced by ETFDH deficiency. Finally, a cell-based model for MADD may be advantageous for drug screenings and clinical management of MADD.

## Figures and Tables

**Table 1:** Acylcarnitine profile analysis from patients with multiple acyl-coenzyme A (CoA) dehydrogenase deficiency (MADD).

Test	Patient 1 (µmol/L)	Patient 2 (µmol/L)	Reference Range (µmol/L)
Alanine	165.93	182.88	<409.20
Arginine	6.55	2.49	<16.57
Citrulline	10.99	9.53	3.21–19.50
Glycine	71.07	62.83	<431.08
Leucine/Isoleucine	144.03	115.21	36.59–185.76
Methionine	19.93	18.56	8.11–27.49
Ornithine	16.15	9.86	<53.40
Phenylalanine	49.46	37.33	33.61–81.56
Tyrosine	41.59	24.97	<84.40
Valine	162.68	163.22	<283.37
Free carnitine	36.14	12.22	13.34–53.92
C2-acylcarnitine	12.11	5.75	8.03–31.02
C3-acylcarnitine	1.60	0.50	<4.01
C3DC-acylcarnitine	0.09	0.09	<0.27
C4-acylcarnitine	0.57	0.50	<0.62
C4DC-acylcarnitine	0.33	0.24	<0.37
C5-acylcarnitine	0.40	0.58	<0.2
C5DC-acylcarnitine	0.02	0.03	<0.05
C5OH-acylcarnitine	0.20	0.21	<0.59
C6-acylcarnitine	0.46	0.21	<0.32
C8-acylcarnitine	0.56	0.33	<0.31
C8:1-acylcarnitine	0.18	0.05	<0.41
C10-acylcarnitine	0.88	0.32	<0.3
C10:1-acylcarnitine	0.23	0.14	<0.45
C12-acylcarnitine	1.18	0.62	<0.39
C12:1-acylcarnitine	0.14	0.22	<0.15
C14-acylcarnitine	0.55	0.52	<0.22
C14:1-acylcarnitine	0.54	0.86	<0.32
C14OH-acylcarnitine	0.01	0.01	<0.09
C16-acylcarnitine	1.42	1.66	0.27–1.87
C16:1-acylcarnitine	0.40	0.68	<1.13
C16OH-acylcarnitine	0.01	0.04	<0.1
C18-acylcarnitine	1.07	1.18	0.18–1.47
C18:1-acylcarnitine	1.13	1.45	0.18–2.15
C18OH-acylcarnitine	0.03	0.04	<0.11
C3/C2	0.13	0.09	<0.27
C5DC/C16	0.02	0.02	<0.19
C8/C10	0.63	1.06	<0.53
Phe/Tyr	1.19	1.49	<2.82
C0/(C16+C18)	14.51	4.30	7.65–48.65
C4OH	0.00	0.00	<0.53
C5:1	0.00	0.00	<0.25

C0, free carnitine.
